# Diagnostic value of volumetric capnography in patients with chronic cough variant asthma

**DOI:** 10.6061/clinics/2020/e1662

**Published:** 2020-10-05

**Authors:** Xiaoli Sun, Wenlan Yang, Sugang Gong, Shuo Liang, Shuyi Gu, Haiwen Lu, Jinmin Liu, Jinfu Xu

**Affiliations:** I Department of Respiratory and Critical Care Medicine, Shanghai Pulmonary Hospital Tongji University School of Medicine, Shanghai 200433, China; II Department of Pulmonary Function Test, Shanghai Pulmonary Hospital Tongji University School of Medicine, Shanghai 200433, China; III Department of Cardio-Pulmonary Circulation, Shanghai Pulmonary Hospital Tongji University School of Medicine, Shanghai 200433, China

**Keywords:** Cough Variant Asthma, Pulmonary Function Test, Volumetric Capnography, Dead Space

## Abstract

**OBJECTIVES:**

To evaluate the quantitative changes and diagnostic performance of volumetric capnography (VCap) parameters in patients with cough variant asthma.

**METHODS:**

This cross-sectional study enrolled 31 patients with cough variant asthma and 30 patients with chronic cough without asthma between November 2010 and March 2012. VCap measurements were recorded at baseline, during the five steps of the histamine challenge, and after bronchodilation with salbutamol. They were then compared between the baseline and histamine challenge, and between the two groups. Receiver operating characteristic curve analysis was performed for different VCap measurements.

**RESULTS:**

The slope of phase III (dc\dv3) and the ratio of phase III slope to phase II slope (SR23%) decreased from baseline upon challenge with 1.1 mg histamine in cough variant asthma patients but increased in patients with chronic cough without asthma. Additionally, the change upon challenge with 1.1 mg histamine in dc\dv3 from baseline (S6-S1dc\dv3) in cough variant asthma patients had the largest area under the curve (AUC) (0.814, 95% CI: 0.697-0.931; *p*<0.001). The AUC for change upon challenge with 1.1 mg histamine in SR23% from baseline was 0.755 (95%CI: 0.632-0.878; *p*<0.001). At a cutoff of 19.8, S6-S1 dc\dv3 had a sensitivity of 74.2% and specificity of 90.0% and at a cutoff of 40.7, S6-S1 SR23% had a sensitivity of 48.4% and specificity of 96.7%.

**CONCLUSION:**

Patients with cough variant asthma exhibit distinct VCap responses for dead space parameters upon challenge with histamine in comparison to patients with chronic cough. VCap parameters like phase III slope and phase III/phase II slope ratio could be used to aid the diagnosis of cough variant asthma.

## INTRODUCTION

Asthma is the most common chronic pulmonary disease among children and young adults globally. It is a major cause of morbidity and poor quality of life and poses a serious burden on the global health care systems (1). Cough variant asthma is a type of asthma with main presentations of dry, non-productive cough and chest tightness, but lacks classic asthma symptoms such as wheezing or shortness of breath (2). Its worldwide prevalence is estimated between 1-18% (3,4). The main treatment for cough variant asthma include bronchodilators and corticosteroids (5). However, because of its atypical clinical manifestations, patients are frequently misdiagnosed with bronchitis and receive inappropriate treatment, which could lead to a gradual decline in pulmonary functions and decreased physical activity (6,7). Thus, accurate diagnosis and prompt treatment are important for cough variant asthma patients. Typically, spirometry is required for confirmation of asthma diagnosis. However, spirometry is an effort-dependent test and requires adequate cooperation from the patient (8).

During the expiratory phase, CO_2_ content increases as the proportion of alveolar gas rises and peaks at the end of expiration. Volumetric capnography (VCap) traces the ratio of CO_2_ concentration in the expiratory gas and detects changes of expired CO_2_ content during the breathing cycle (9). Bronchoconstriction leads to a reduction in the volume of conducting airways and an increase in alveolar dead space, which can be detected by VCap (10). Studies have shown that VCap could be used as a quantitative method to diagnose and evaluate pulmonary functions in patients with chronic respiratory diseases, such as asthma or chronic obstructive pulmonary disease (11,12). Previously, we also demonstrated that VCap could be applied to measure dynamic changes of airway dead space with bronchodilator use in healthy subjects (13). However, its role in the diagnosis and evaluation of pulmonary functions in patients with cough variant asthma is unclear and has not been studied.

In the current double-blind cross-sectional study, we examined chronic cough patients with or without asthma by VCap and determined the quantitative changes and diagnostic value of VCap in patients with cough variant asthma.

## MATERIALS AND METHODS

### Study design and participants

This double-blind cross-sectional study recruited chronic cough patients between November 2010 and March 2012. Asthma was diagnosed according to the Global Initiative for Asthma (GINA2019) guidelines (14) and confirmed by spirometry and was differentiated from chronic cough lasting ≥8 weeks without obvious evidence of lung disease on X-ray chest film. Asthma was confirmed by spirometry, upon bronchoprovocation, and by measuring forced vital capacity (FVC). The diagnosis of asthma was confirmed if 1) the positive bronchial provocation test; 2) the positive bronchodilation test (after inhalation of bronchodilator, FEV_1_ increased by >12%, and forced expiratory volume in 1 second (FEV_1_) increased by an absolute value of >200 mL; 3) daily variations between day and night time in peak expiratory flow (PEF) (at least 7 consecutive days, daily PEF day and night variability rate/7) >10%, or weekly variation rate of PEF [(highest PEF in 2 weeks-lowest PEF)/[(2 weeks maximum PEF+lowest PEF)×1/2]×100%] >20%; and 4) a reduced ratio FEV_1_ to FVC was identified. The main inclusion criteria were as follows: 1) patients with chronic cough ≥8 weeks; 2) patients suspected of cough variant asthma by the treating physician; 3) FEV_1_ >70% predicted; and 4) patients able to follow instructions and complete VCap. The major exclusion criteria were as follows: 1) a history of smoking; 2) previous diagnosis of asthma, allergic rhinitis, or eczema; 3) acute respiratory tract infection; 4) history of allergy to histamine; 5) patients with cardiac dysfunction, aortic aneurysm, hypertension, or hyperthyroidism; and 6) pregnant patients.

The study protocol was approved by the local ethics committee of the hospital. All study participants provided written informed consent. The study was conducted in accordance with the Declaration of Helsinki.

### Volumetric capnography

Volumetric capnography (VCap) was performed as described previously (13). Briefly, long-acting β2-agonists, antihistamines, and oral corticosteroids were discontinued 48h before the test. Short-acting β2-agonists, anticholinergics, and leukotriene receptor antagonists were discontinued 24h before the test. Oral theophylline was discontinued 12-48h before the test. Inhaled corticosteroid was discontinued 12h before the test. Patients were also instructed to avoid strenuous exercise and exposure to cold air and were asked not to have coffee, tea, cola drinks, chocolate, and other caffeinated foods before the test.

The histamine provocation test was done using the automatic APS Aerosol Excitation System (Jaeger, Wurzburg, Germany), which uses computerized precise drug administration. The APS atomizer was connected to a compressed air source and the mouthpiece at the two ends with compressed air pressurized at 3.5 kg/cm^2^. The flow rate was subjected to a spray discharge flow rate of 0.13 mL/min. Two histamine concentrations, 2.5% and 5%, were used. The test was performed in six steps (Table 1) and baseline values of FEV_1_ and the PEF were measured prior to drug inhalation. The subjects inhaled normal saline at baseline and then graduated steps of histamine with 0.07 mg histamine as an initial dose. FEV_1_ and PEF were measured for 1 min to 3 min in each step after inhalation of the drug. The interval between doses was 5 min. If FEV_1_ decreased by <20% from baseline, the subject continued to receive the next dose of histamine until a cumulative dose reached 2.2 mg. The test was terminated if the subjects showed either severe dyspnea or FEV_1_ was reduced by ≥20% from baseline or the highest dose of histamine was reached followed by salbutamol to reverse the process. Medications used during the six steps of VCap test are listed in Table 1.

**Figure 1:**
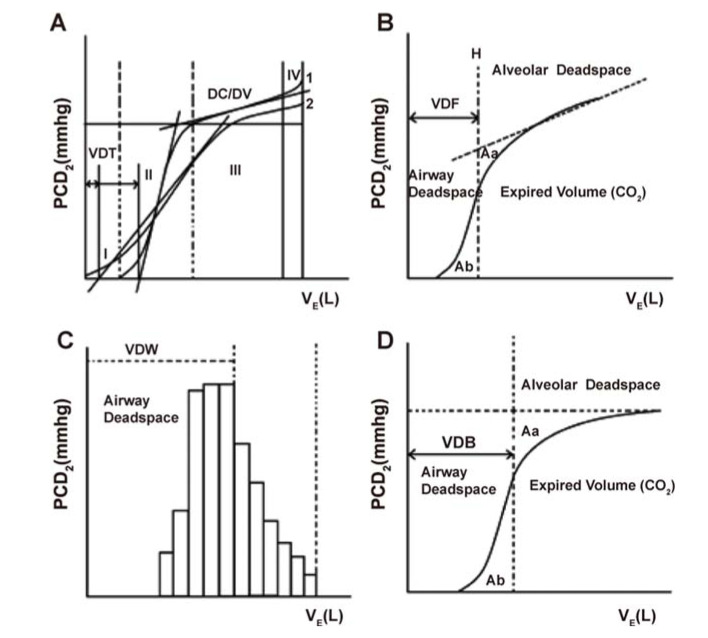
Illustrative phases and dead spaces on waveform patterns of capnovolumetry (PCO_2_/VE Plot). **(A)** Phase I is the CO_2_-free ineffective Vt and a series of dead spaces including Threshold dead space (VDT). Phase II represents the transition between the airway and alveolar gas. Phase III contains the alveolar plateau (dC3/dV calculated by its slope formula) and is the CO_2_ containing part of the breath. Phase IV represents a period of acceleration in CO_2_ concentration. Curve 1 represents VCap diagram and VDT at the tidal volume prior to the provocation test. Curve 2 represents VCap and VDT following the Ach challenge. **(B)** Fowler dead space (VD-F). PCO_2_: expiratory CO_2_ partial pressure, VE: expiratory volume. The approximately straight line of phase III is first reversed and extended, and then a straight line (H) is drawn parallel to the longitudinal axis, which intersects the extension cord of phase III, the curve of phase II, and the horizontal axis. Thus, the areas of the approximate triangles Aa and Ab are equal. The expiratory volume corresponding to the intersection of line H with the horizontal axis is the dead space volume. **(C)** Wolff dead space (VD-W). This curve is divided into several small broken lines. The slope of the small line (ΔPCO_2_/ΔV) is calculated, and drawn on the volume axis to obtain the volume distribution function. The expiratory volume corresponding to the average of the function is VD-W. **(D)** Bohr dead space (VD-B). A horizontal parallel line is drawn on the CO_2_ concentration at the end of the breath, and a straight line is made parallel to the longitudinal axis to equalize the Aa and Ab areas. The expiratory volume corresponding to the intersection of this straight line with the horizontal axis is the dead space volume.

Detailed measurement methods and calculations of VCap values, including dead space volumes [Fowler dead space (VD-F), Wolff dead space (VD-W), threshold dead space (VD-T), Bohr dead space (VD-B)], were the same as described previously (13). Briefly, under calm breathing, VCap was undertaken in three phases (Figure 1) with exhaled CO_2_ concentration or partial pressure shown on the vertical axis in the capnogram. In the first phase, which is the baseline, exhaled breath is pure anatomical dead space gas without CO_2_. The approximate straight line in phase I is extended in reverse and intersects with the horizontal axis. VD-T is the volume of exhaled breath corresponding to the intersection (Figure 1A). The second phase is the expiratory phase when the alveolar gas is mixed with the airway gas and is represented by the ascending portion of a straightened S-shaped curve. The approximate straight line in phase II firstly extends and then is parallel to the vertical axis and intersects with VCap phase III extension line into the VCap phase II curve and the horizontal axis, respectively. There are equal areas between two approximate triangles Aa and Ab. VD-F is the volume of exhaled air corresponding to the intersection of this line and the horizontal axis (Figure 1B). The third phase is the late expiratory phase with a high CO_2_ concentration in the alveolar gas and is represented by a horizontal plateau in the capnogram. A line parallel to the vertical axis is drawn at twice the exhaled breath volume (H) corresponding to half the exhaled breath concentration. The volume CO_2_ curve portion intercepted by the line is analyzed, which is divided into a plurality of small fold lines. The slope of each small fold line (△PCO_2_/ΔV) is calculated. The volume axis is drawn to obtain the slope volume distribution function. VD-W is the volume of the exhaled breath corresponding to the average value of this function (Figure 1C). In addition, a parallel horizontal axis is drawn at the end of expiratory CO_2_ concentration. Then, a straight line, parallel to the vertical axis, is drawn to make equal areas between Aa and Ab. VD-B is the volume of exhaled air corresponding to the intersection of the line and the horizontal axis (Figure 1D).

### Outcome measurements

Pulmonary ventilation function measurements included FVC, FEV_1_, and maximum PEF. VCap measurements were end-tidal CO_2_ concentration (CO_2_et), phase II slope (dC2) /DV), phase III slope (dC3/DV), ratio of phase III to phase II slope (SR23), and different dead space calculations.

### Statistical analysis

All continuous data were presented as mean±standard deviation, and compared by the Student’s *t*-test or Wilcoxon two-sample test, where appropriate. All categorical data are presented as percentage and compared by Chi-square analysis. Receiver operating characteristic curve analysis was performed for different VCap measurements. Areas under the curve (AUCs) were compared by the Mann-Whitney test. Cutoff values were determined at the maximum value for Yonden's index. A *p-*value <0.05 was considered statistically significant. Statistical analysis was done using SPSS (Version 9.3, SPSS, IBM, Chicago, USA).

## RESULTS

A total of 61 patients were enrolled in the current study, with 31 patients diagnosed with cough variant asthma and 30 patients diagnosed with chronic cough without asthma. Their baseline characteristics are listed in Table 2. Patients with cough variant asthma had statistically significantly higher weight and body mass index compared to patients with chronic cough. The two groups were comparable in demographic and other baseline variables.

**Figure 2:**
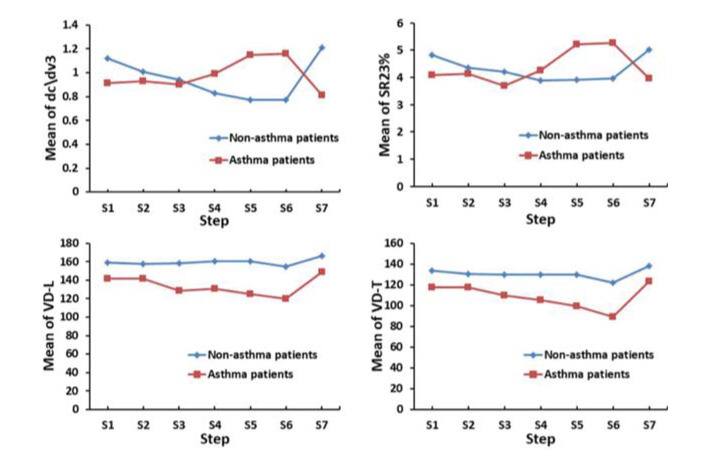
General linear model-repeated measures trend test for S1 to S7 in patients with cough variant asthma or chronic cough.

Table 3 and Figure 2 show VCap measurements in patients with cough variant asthma or chronic cough. The dead space measurements at baseline including VD-W, VD-F, VD-L, and VD-T were significantly higher in patients with chronic cough than in those with cough variant asthma (*p*<0.05). Significantly greater reduction from baseline in VD-T was observed in patients with cough variant asthma *versus* those with chronic cough upon challenge with 0.205, 0.825, and 1.1 mg histamine (*p*<0.05). Significantly greater reduction from baseline in VD-L was also observed in patients with cough variant asthma than in those with chronic cough upon challenge with 0.825 and 1.1 mg histamine (*p*<0.05). There was a significantly greater increase from baseline in tidal volume (V_T_) in patients with chronic cough than in those with cough variant asthma upon challenge with 1.1 mg histamine (*p*=0.036). Furthermore, the slope of phase III (dc\dv3) decreased from baseline upon challenge with 1.1 mg histamine in patients with chronic cough while it increased in those with cough variant asthma (*p*<0.001). In addition, the ratio of phase III slope to phase II slope (SR23%) decreased from baseline upon challenge with 1.1 mg histamine in patients with chronic cough while it increased in patients with cough variant asthma (*p*=0.005). These dynamic changes in dead space measurements indicated that patients with chronic cough and those with cough variant asthma showed distinct responses upon challenge with histamine.

**Figure 3:**
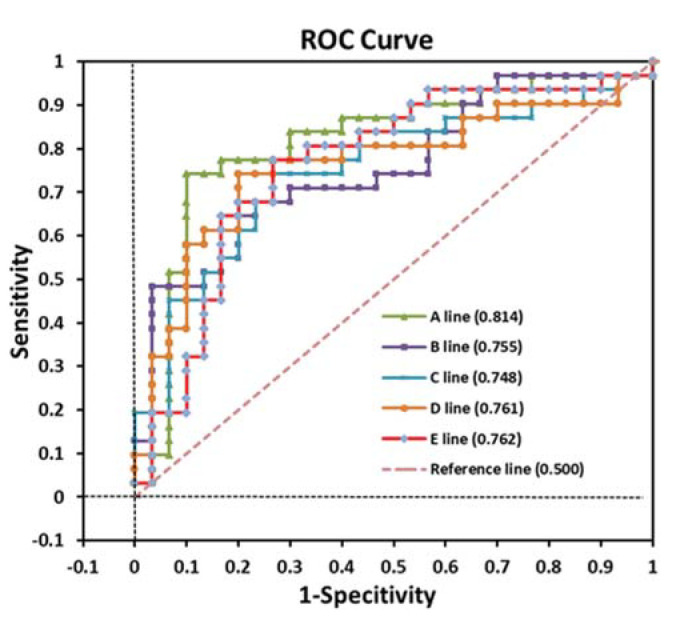
Area under the curve analysis. A line: S6-S1 dc\dv3; B line: S6-S1 SR23%, C line: S5-S1 VD-L, D line: S5-S1 VD-T; E line: S6-S1 VD-T.

Tables 4 and 5 and Figure 3 show the diagnostic performance of VCap in cough variant asthma assessment. The AUC of positive histamine challenge test was 0.871 (95%CI: 0.791-0.949; *p*<0.001). The change upon challenge with 1.1 mg histamine in the slope of phase III from baseline (S6-S1dc\dv3) in cough variant asthma patients had the largest AUC (0.814, 95%CI: 0.697-0.931; *p*<0.001) followed by change upon challenge with 0.825 or 1.1 mg histamine from baseline in V_T_ (0.761, 95%CI: 0.634-0.889 and 0.762, 95%CI: 0.636-0.899, respectively; *p*<0.001 in both) and by change upon challenge with 1.1 mg histamine in the ratio of phase III slope to phase II slope (SR23%) from baseline (0.755, 95%CI: 0.632-0.878; *p*<0.001). At a cutoff of 19.8, S6-S1 dc\dv3 had a sensitivity of 74.2% and specificity of 90.0%. At a cutoff of 40.7, S6-S1 SR23% had a sensitivity of 48.4% and specificity of 96.7%.

## DISCUSSION

This double-blind cross-sectional study demonstrates that patients with cough variant asthma exhibit distinct VCap responses for dead space parameters upon challenge with histamine in comparison to patients with chronic cough. VCap revealed significantly greater reduction from baseline in VD-T and VD-L in patients with cough variant asthma than in those with chronic cough upon histamine challenge. The slope of phase III (dc\dv3) and the ratio of phase III slope to phase II slope (SR23%) decreased from baseline upon histamine challenge but increased in patients with cough variant asthma. Furthermore, VCap parameters S6-S1dc\dv3 and SR23% have very high sensitivity and satisfactory specificity. Our findings show that VCap parameters like phase III slope and phase III/phase II slope ratio could be used to aid the diagnosis of cough variant asthma.

Cough variant asthma is a chronic respiratory condition. Its diagnosis might be missed because of its atypical clinical presentations, which could result in slow pulmonary function deterioration. Its accurate diagnosis is essential for relieving clinical symptoms and improving pulmonary function. Previous studies have shown the diagnostic value of VCap in evaluating asthma and chronic obstructive pulmonary disease (11,12). Cough variant asthma has a feature common to other types of asthma, which is increased airway dead space (15). The dynamic change of the airway dead space was considered to be controlled by the constriction and relaxation of the airway smooth muscle (16). In the laboratory, the constriction or relaxation of the airway smooth muscle could be induced by airway stimulating agents, such as histamine or salbutamol (17). The airway dead space and its dynamic changes could be readily measured by VCap as demonstrated in our previous study (13). In the current study, we demonstrated that VCap could be used to assess pulmonary function in patients with cough variant asthma. It also added diagnostic accuracy to this clinical condition.

Bronchial provocation test is a method for detecting airway smooth muscle contraction by chemical, physical, biological, and other artificial stimulation. It is to determine whether the bronchus is narrowed by measuring changes in lung function indices. It is the most common and accurate method to detect airway hyper responsiveness. Clinical examination is one of the important diagnostic criteria for atypical bronchial asthma or cough variant asthma. It is helpful in the diagnosis and differential diagnosis of asthma. It also helps to assess the severity of the disease and evaluate treatment outcomes. It can be used for the study of the pathogenesis of airway diseases. Since it is difficult to apply the bronchial provocation test to directly measure airway diameter, lung function indices are commonly used in clinical practice to reflect changes in airway function. By administering a bronchodilator drug, a bronchodilation test can be performed. However, the airway hyper responsiveness of individual subjects is not the same as the severity of asthma. Airway hyper responsiveness can also be seen in chronic bronchitis and smokers. Therefore, recent asthma symptoms combined with airway hyper responsive testing results are the strongest evidence to diagnose bronchial asthma.

Our previous study on healthy volunteers showed that VCap could be used to evaluate pulmonary function and airway reactivity after bronchodilator administration (13). Dead space, and notV_T_, changes correlated more with airway reactivity. When small airway constriction was induced during the histamine provocation test, dynamic changes of the airway dead space were detected by VCap. This provided a quantitative measurement to evaluate the airway dead space.

Asthma is a condition with small airway constriction and obstruction during acute exacerbations (18). Patients with asthma are more sensitive to inhaled irritants which can cause bronchial hypersensitivity reactions (19). This characteristic hypersensitive reaction was used to diagnose asthma (20). In the current study, we tested the application of VCap in patients with cough variant asthma. Our results showed that dynamic changes in airway dead space were more significant in patients with cough variant asthma compared to patients with chronic cough. These VCap measurements for airway dead space could be used to distinguish patients with cough variant asthma from patients with chronic cough.

Previously, Abid et al. reported a model to use VCap to diagnose obstructive lung diseases (21). In the current study, we also tested the performance of VCap to diagnose cough variant asthma. We found that the histamine challenge test had a good AUC value (0.871, 95%CI: 0.791-0.949), which indicated that it had a better diagnostic performance compared to salbutamol bronchodilator test when used alone. With the challenge test under different doses of histamine, we tested the diagnostic performance of different dynamic measurements. We found that the highest sensitivity was observed when the cutoff of S6-S1 dc\dv3 was set at 19.8, and the highest specificity was observed when the cutoff of S6-S1 SR23% was set at 40.7. This suggested that dynamic changes in the dead space with an increasing dose of histamine could be used to diagnose cough variant asthma. This result should be confirmed by future studies. In addition, the diagnostic performance of the test, which combines the histamine challenge test, salbutamol bronchodilator test, and clinical presentations, should also be investigated.

Patients with asthma require close clinical follow-ups and monitoring for disease progression. Adjustments of the treatments are required to improve the clinical outcomes (22). One previous study showed that VCap could be used for repeat measurements of the airway dead space to determine the treatment responses (11). In future studies, we plan to apply the quantitative measurements identified in the current study to monitor the treatment responses in patients with cough variant asthma and evaluate whether these measurements could be used as markers to guide clinical treatments.

Limitations for our study included the small number of patients and lack of a widely accepted, standard diagnostic criterion for cough variant asthma. We enrolled patients with clinical suspicions for cough variant asthma, which would limit the generalizability of our study.

In conclusion, we tested the application of VCap in the diagnosis and evaluation of pulmonary functions in patients with cough variant asthma. Dead space variations could be used to distinguish cough variant asthma from chronic cough. VCap histamine challenge test can be applied in the clinic to diagnose cough variant asthma.

## AUTHOR CONTRIBUTIONS

Sun X and Yang W prepared the manuscript. Gong S and Liang S collected the data. Gu S and Lu H performed the data analysis. Liu J provided resources. Xu J designed the study.

## Figures and Tables

**Table 1:** Six steps during volumetric capnography (VCap) measurements.

Step	Interval (min)	Concentration	Dose	Cumulative Dose	Substance
S1	0	-	-	-	-
S2	3	0.9%	0.072 mg	0.0562 mg	Normal saline
S3	3	4 mg/mL	0.007 mg	0.07 mg	Histamine
S4	3	4 mg/mL	0.205 mg	0.275 mg	Histamine
S5	3	32 mg/mL	0.825 mg	1.1 mg	Histamine
S6	3	32 mg/mL	1.1 mg	2.2 mg	Histamine
S7	15-20	100 μg/Puff	3 Puffs	-	Salbutamol

-, none.

S1, baseline value of FEV_1_ and peak expiratory flow prior to inhalation of the drug.

S2, subjects inhaled saline.

S3-S6, subjects inhaled different doses of histamine.

S7, subjects inhaled salbutamol after the bronchial challenge.

**Table 2:** Patient baseline characteristics*.

Variables	Chronic cough (N=30)	Cough variant asthma (N=31)	*p*
Age, years	39.9±11.47	40.77±12.67	0.723
Height, cm	162.33±6.88	162.68±7.64	0.854
Body weight, kg	60.43±9.43	67.16±9.79	0.010
BMI, kg/m^2^	22.89±2.9	25.36±3.29	0.003
Male sex, N (%)	11 (36.67)	14 (45.16)	0.500

* Data are expressed as mean±standard deviation unless otherwise indicated.

BMI, body mass index.

**Table 3:** Pulmonary function evaluation by VCap in patients with chronic cough or cough variant asthma.

Variables	Chronic cough (N=30)	Cough variant asthma (N=31)	*p*
S1 dc\dv2 (%/L)	22.71±7.28	23.36±5.51	0.512
S1 dc\dv3 (%/L)	1.12±0.94	0.91±0.5	0.579
S1 SR23% (%)	4.83±3.4	4.08±2.4	0.292
S1 CO_2_et (mmHg)	3.87±0.73	3.79±0.5	0.681
S1 VD-B (mL)	277.07±56.03	264.1±55.94	0.189
S1 VD-W (mL)	221.8±38.57	196.61±37.29	0.012
S1 VD-F (mL)	213.67±38.42	187.94±32.55	0.006
S1 VD-L (mL)	159.27±33.85	141.39±27.21	0.026
S1 VD-T (mL)	133.4±27.27	117.74±25.55	0.024
S1 V_T_ (L)	0.9±0.25	0.97±0.28	0.318
S6-S1 dc\dv3 (%/L)	-13.03±43.99	23.04±41.59	<0.001
S6-S1 SR23% (%)	-6.82±39.75	39.91±66.25	0.005
S5-S1 VD-L (mL)	2.23±14.46	-11.39±16.72	0.001
S6-S1 VD-L (mL)	-1.97±14.95	-14.62±17.44	0.005
S4-S1 VD-T (mL)	-0.84±14.72	-9.82±16.54	0.010
S5-S1 VD-T (mL)	-1.17±14.88	-15.57±14.24	<0.001
S6-S1 VD-T (mL)	-7.43±15.48	-23.4±16.07	<0.001
S6-S1 V_T_ (L)	12.15±29.43	1.24±31.74	0.036

S1-7 represent six steps during VCap measurements. S6-S1 represent changes for a measurement from S1 to S6. The degree of changes was calculated as (S6-S1)/S1 * 100%.

Fowler dead space (VD-F), Wolff dead space (VD-W), threshold dead space (VD-T), Bohr dead space (VD-B), Langley dead space (VD-L ), the slop of phase III (dc\dv3), the slop of phase II (dc\dv2) Ratio of phase III slope to phase II slope (SR23%), tidal volume (V_T_), end-tidal carbon dioxide (CO_2_et).

**Table 4:** Diagnostic performance of VCap in chronic cough variant asthma.

	AUC				Difference from challenge test AUC			
Variables	AUC	95%CI		*p* a	AUC	95%CI		*p* b
Positive challenge test	0.871	0.793	0.949	<0.0001				
Weight	0.693	0.557	0.828	0.006	-0.179	-0.323	-0.034	0.015
BMI	0.714	0.583	0.845	0.001	-0.157	-0.301	-0.013	0.033
S1 FEV_1_	0.584	0.437	0.732	0.261	-0.287	-0.466	-0.107	0.002
S1 PEF	0.653	0.511	0.794	0.035	-0.218	-0.397	-0.040	0.016
S1 FEV_1_/FVC	0.723	0.595	0.850	0.001	-0.148	-0.298	0.001	0.051
S1 dc\dv2	0.550	0.402	0.697	0.511	-0.322	-0.497	-0.146	0.000
S1 dc\dv3	0.542	0.394	0.690	0.580	-0.329	-0.491	-0.168	<0.001
S1 SR23%	0.579	0.432	0.726	0.293	-0.292	-0.455	-0.129	0.001
S1 CO_2_et	0.531	0.381	0.681	0.684	-0.340	-0.503	-0.177	<0.001
S1 Vm_25-50_	0.531	0.383	0.678	0.684	-0.340	-0.517	-0.163	0.000
S1 VD-B	0.598	0.451	0.746	0.192	-0.273	-0.450	-0.095	0.003
S1 VD-W	0.696	0.558	0.834	0.005	-0.175	-0.340	-0.010	0.038
S1 VD-F	0.677	0.541	0.813	0.011	-0.194	-0.350	-0.039	0.014
S1 VD-L	0.668	0.530	0.807	0.017	-0.203	-0.367	-0.039	0.015
S1 VD-T	0.669	0.532	0.805	0.015	-0.202	-0.365	-0.040	0.015
S1 V_T_	0.545	0.398	0.693	0.548	-0.326	-0.486	-0.166	<0.0001
S6-S1 dc\dv3	0.814	0.697	0.931	<0.0001	-0.057	-0.186	0.072	0.385
S6-S1 SR23%	0.755	0.632	0.878	<0.0001	-0.116	-0.257	0.025	0.107
S5-S1 VD-L	0.748	0.621	0.876	0.000	-0.123	-0.266	0.021	0.093
S6-S1 VD-L	0.724	0.591	0.856	0.001	-0.147	-0.294	-0.001	0.049
S4-S1 VD-T	0.691	0.555	0.828	0.006	-0.180	-0.334	-0.025	0.022
S5-S1 VD-T	0.761	0.634	0.889	<0.0001	-0.110	-0.247	0.028	0.118
S6-S1 VD-T	0.762	0.636	0.889	<0.0001	-0.109	-0.250	0.033	0.132
S6-S1 V_T_	0.693	0.557	0.828	0.005	-0.179	-0.327	-0.030	0.018

a compared to the reference line AUC=0.5.

b compared to the AUC from the challenge test.

Fowler dead space (VD-F), Wolff dead space (VD-W), threshold dead space (VD-T), Bohr dead space (VD-B), Langley dead space (VD-L ), the slop of phase III (dc\dv3), the slop of phase II (dc\dv2) ratio of phase III slope to phase II slope (SR23%), tidal volume (V_T_), end-tidal carbon dioxide (CO2et) , forced vital capacity in one second (FEV_1_ ), maximum expiratory flow rate (PEF), ratio between forced vital capacity in 1 second and forced vital capacity ( FEV_1_/FVC), capacity with exhaled carbon dioxide concentration between 25% and 50% (Vm25-50).

**Table 5:** Sensitivity and specificity of VCap challenge test.

Variables	Cutoff	Sensitivity	Specificity
S6-S1 dc\dv3	19.8	0.742	0.900
S6-S1 SR23%	40.7	0.484	0.967
S5-S1 VD-L	-4.3	0.733	0.742
S5-S1 VD-T	-6.13	0.733	0.774
S6-S1 VD-T	-13.7	0.733	0.774
